# The Garden and Landscape as an Interdisciplinary Resource Between Experimental Science and Artistic–Musical Expression: Analysis of Competence Development in Student Teachers

**DOI:** 10.3389/fpsyg.2020.02163

**Published:** 2020-09-04

**Authors:** Amparo Hurtado-Soler, Pablo Marín-Liébana, Silvia Martínez-Gallego, Ana María Botella-Nicolás

**Affiliations:** ^1^Department of Experimental and Social Sciences Teaching, Faculty of Teacher Training, Universitat de València, Valencia, Spain; ^2^Department of Musical, Visual, and Corporal Expression Teaching, Faculty of Teacher Training, Universitat de València, Valencia, Spain

**Keywords:** preservice teachers, organic learning gardens, teachers’ competencies, sound landscape, TICs, interdisciplinary

## Abstract

The garden is a major educational resource that can be used for all areas of knowledge from an interdisciplinary perspective as it reflects the complexity and interactions of the natural environment. This research was carried out in the academic years 2017–2018 and 2018–2019 with 418 participants of from the second, third, and fourth year of the Degree in Primary Education at the University of Valencia. The use of the garden and the landscape is analyzed as a non-formal context for training primary school teachers through sensory experiences that contribute to the development of scientific and artistic competencies. The study focused on the relationship between natural sounds and emotions to promote the environmental awareness and active listening that are necessary to improve auditory perception and reflect on health problems caused by noise pollution. During the project, we discovered the synergies between experimental sciences and artistic–musical expression and their importance for developing university students’ competencies in the professional, personal, and collective spheres. The activities consisted of (1) a walking tour from the Faculty of Education to the garden at 1 km from the center, to reflect on the origin and characteristics of sound, emotions and their relationship with music; (2) sensory experiences in the garden through visual and auditory interaction with the landscape; and (3) an immersion in quasi-real scenarios created by virtual reality. The analysis was carried out by means of an *ad hoc* questionnaire with Likert-type items focused on aspects of the usefulness of gardens and the activities involved in the general and specific skills of the degree and open questions for reflections on emotional health, environmental sustainability, and artistic–musical creation as a representation of the environment. The results show the importance of the garden as a resource for training of primary teachers in a holistic and global approach that contributes to the development of teaching skills and the acquisition of knowledge based on sensory experiences with the landscape.

## Introduction

Social, scientific, and technological changes in the twenty-first century have modified educational systems and shown the need for quality education at all levels and social contexts. This new vision of teaching should be directed toward the development and training of committed, free, and critical citizens able to achieve a sustainable world (UNESCO, 2018). We must therefore question the formation of future teachers and the expectations of the teaching methods they receive (Porto et al., 2018). Teacher training should be based on a constructionist approach and directed toward asking questions about the ideal learning method and how we should teach in order to favor the process of changing ideas (Furió, 1994; Sanmartí et al., 2002).

This transformation should consider not only knowledge as regards knowing and knowing how to (concepts and procedures) but also personal qualities (knowing how to be) regarding decision making and exchanging information, which will be the basis of the future effective professional activities (Esteve and Alsina, 2010; Alsina, 2013). In other words, learning must be constructive and creative and produce learning that allows teachers in training achieve the necessary professional competencies for teaching (Hurtado et al., 2018b). Initial teacher training should thus become an educational revolution that facilitates change and renovation from an early age with the aim of improving the teaching–learning process through research–action using the multiple resources of formal and non-formal education. According to Bolarín and Moreno (2015), we must promote an approach to reality to achieve deep and constructive learning that allows students to go on learning permanently. This means we must design consistent situations and teaching practices that include the learning of contents, teaching strategies, and the students’ ideas (Confederación de Sociedades Científicas de España [COSCE], 2011).

The educational garden and the landscape are resources that can improve students’ perception of the complexity and systematic methods of nature (Pérez-López et al., 2020). They are multidisciplinary learning spaces that include both manipulative activities (Bredderman, 1982; Waliczek and Zajicek, 1999) and intellectual activities (Klemmer et al., 2005) and facilitate the connection between scientific and artistic disciplines (Botella and Hurtado, 2016, 2017). In this context, carrying out activities in the natural environment can favor the integration of theory and practice (Caamaño, 2003), because they can address scientific problem solving (Del Carmen, 2011) and thus contribute to improving academic performance (Blair, 2010; Williams and Dixon, 2013) and teaching skills (Cantó et al., 2013; Muñoz and Carmona, 2017). Also, the contact with nature is associated with human well-being (Zhang et al., 2014; Sobko et al., 2018). This promotes the acquisition of sustainable attitudes and environmental responsibility (Zelenski et al., 2015; Aragón, 2017; Evans et al., 2018) and facilitates cooperative learning, social integration, equality, and solidarity (Ozer, 2006; Cantó et al., 2016; Eugenio and Aragón, 2016; Botella et al., 2017).

Working in the educational garden promotes the development of an educational practice that mostly involves a combination of three dimensions (Botella et al., 2014).

### Educate in the Environment

Through manipulation, experiences, and investigation in the environment that helps an understanding of the relationships between local and global problems.

### Educate About the Environment

Analyzing each of the environmental elements and the relationships between those human and those natural. In this way, the garden becomes an ecological, social, and cultural system where everything is interconnected.

### Educate in Favor of the Environment

Promoting the acquisition of values and attitudes toward the environment with the environment and drive a change in order to achieve a most responsible and sustainable behavior.

Many students consider that outdoor classrooms help well-being and class participation (Kuo et al., 2018; Largo-Wight et al., 2018), whereas lack of interaction with the environment can reduce positive attitudes and prevent emotional perceptions related to health and welfare (Soga and Gaston, 2016). Learning in a garden can be approached from a sensorial aspect to get to know the elements in a landscape and the emotions they transmit (Botella et al., 2018). The easily perceptible set of components in the ecosystem such as size, proportion colors, smells, and sounds is known as the *fenosystem* (Costa, 2013). A study by Sun et al. (2018) suggests that sound and vision interact, although the latter dominates in most landscapes.

Even though sounds are just as important as shapes and colors, the scientific exploration of the acoustic environment or soundscape is a recent project. The soundscape [a term coined by Schafer (1967)] is formed by sound parameters (pitch, intensity, tone, and duration) together with noise and silence. Every combination of sound and noise forms part of a specific environment and is a unique soundscape with its own particular identity (Schafer, 2013; Rodríguez, 2015). Listening must be voluntary, whereas hearing refers to the auditory capacity (De la Ossa, 2015). Auditory work promotes observation of the environment because listening to the social and natural surroundings provides knowledge that is not perceived by permanent contact (Akoschky, 1996). The soundscape or acoustic ecology permits the study of the effects of the acoustic landscape on physical responses or the behavioral characteristics of those who live there (EARS, 2014). Their specific objective is to direct the attention to certain discrepancies that may have unfavorable or unhealthy effects. Providing incentives to the auditory senses and conscious listening to different surroundings can help to study the relation between natural sounds and the emotions and promote sensitivity to the environment. It is important for students to feel part of the ecosystem and understand the reasons for certain problems as this makes them search for sensible solutions (Caurín et al., 2012). From this new perspective, synergy can be established with scientific and artistic disciplines using the garden–landscape binomial to reflect on the qualities of sound and environmental problems and how they affect our health.

This preliminary study is presented as part of a larger research process that has been divided into several phases. The main objective of this initial phase is to evaluate the usefulness of garden and landscape as interdisciplinary resources for student teachers to learn and develop scientific and artistic skills. Sensory experiences deepen the relationship between the visual and auditory elements in the garden, the landscape and the emotions, and a study was made of how these experiences can promote environmental awareness, facilitate reflection on noise pollution and health, and establish synergies between artistic and scientific disciplines.

## Materials and Methods

### Participants

A total of 418 students from the second-, third-, and fourth-year Primary Education Teacher’s Degree participated in the study at the University of Valencia in the 2017–2018 and 2018–2019 academic years. The syllabus included six subjects related to sciences, two of which are compulsory, whereas the others are optional specializations: *information and communication technologies*, *science and mathematics*, and *musical education.* From all participants, 294 were female, and 124 were male (Table 1).

**TABLE 1:** Distribution of participants by year, subject, academic year, and specialty.

Academic year	Subject matter	Grade	Itineraries	Participants (*n* = 418	
				Female	Male
2017–2018	NST	2	Compulsory	52	4
	DEM	3	ITC	28	25
	TPSM	3	Science and mathematics	20	5
	TPSM	3	Science and mathematics	18	4
	TCYM	4	Science and mathematics	29	14
	TCYM	4	ITC	13	6
2019–2020	NST	2	Compulsory	31	4
	DEM	3	ITC	20	17
	TPSM	3	Science and mathematics	16	15
	TSEBH	4	Musical education	18	14
	TSEBH	4	Science and mathematics	33	9
	ICTTRSM	4	ITC	16	7

*NST, natural sciences for teachers; DEM, designing educational material; TPSM, teaching proposals for sciences and mathematics; ICTTRSM, ICT as teaching resources in sciences and mathematics; TSEBH, teaching science: environment, biodiversity and health; ITC, information technology communication.*

The learning garden is located 1 km from the campus in a large agricultural area called *Huerta de Valencia* that surrounds the city of Valencia (Spain) and is linked to urban dynamics. This agroecological landscape is unique in Europe and has existed for more than two millennia. The route from the Faculty of Education to the garden crosses this agricultural landscape by pedestrian pathway, so that students can interact with the landscape and observe the differences between urban and agricultural surroundings. The learning garden is composed of an arable area of 2,400 sqm divided into three zones: fruit trees, vegetables, and uncultivated land. There is also an area with tables and benches, an outdoor kitchen, and a machinery store. The channel is irrigated through a system of channels throughout the cultivated areas.

### Procedure

The experience was carried out in three sessions for a total of 6 h: the first (3 h) in the outdoor classroom, the second (2 h) in the classroom, and the third (1 h) online to promote reflection (Table 2). Some activities were individual, and some were done in groups. During the activity, the teacher responsible for the subject led each group. Before the sessions, a preliminary intervention was conducted in which the activities were explained, and the materials for the descriptions and data collection (questionnaires and field notebook) were handed out.

**TABLE 2:** Sequence of activities.

Session	Site	Landscape	Activity	Task	Analysis instrument
Session 1 Garden (3 h)	Route	Faculty (human)	Sound level measurement (dB) Identification and description of sounds Emotional perception Musical recreation of sounds and landscapes	Individual	Q1-T1
		Urban (technological)			
		Garden (agroecological)			Q1-T2 Q1-T3
	Garden	Agroecological	Observation of landscape elements, crops, and state of conservation Visual perception of landscape Reflection on noise pollution and health	Group Individual	Report Q2-C
Session 2 Classroom (2 h)	Classroom	Virtual	Visual and auditory immersion in the garden landscape Comparison between virtual and real visual and auditory elements	Individual Group	Q2-A Debate
Session 3 (1 h)	Online	—	Reflection on use of the garden landscape as educational resource Development of teaching competencies	Individual	Q2-B Q3-A Q3-B

The first session involved walking from the Faculty of Education to the garden. During the tour, three types of landscape were observed: the human landscape (inside the Faculty), the urban landscape (street with traffic), and the agricultural landscape (garden and *Huerta de Valencia*). Listening practice was carried out in each landscape. Ambient sounds, sound intensity, emotions, and musical instruments and styles related to each one were recorded. This activity was implemented individually by using contingency tables as part of an *ad hoc* questionnaire (Q1). Once in the garden, in addition to identifying the sounds and taking sound intensity measurements, descriptions of the crops and the state of conservation of the garden were made, and pictures were taken. This activity was carried out in groups of four people, and at the end of the didactic sequence, each group delivered a report. The problems of noise pollution observed during the outdoor activity and its effect on human health were analyzed (Q2-C).

The second session consisted of a virtual immersion in the garden with virtual reality (VR) glasses using 360° videos recorded *ex profeso* by the teachers. In these recordings, scenes that showed life in the soil, the cultivation of fruit trees (avocados and orange trees), domestic animals (donkey, cats, and dogs), and some agricultural practices (planting and irrigation) were displayed. Access to the videos was through a YouTube channel^1^. After the activity, the use of VR was assessed, and the differences between the real and virtual perception of the landscape were analyzed by an individual questionnaire (Q2-A) and a sharing in the classroom. The descriptions and photographs taken in the garden were used to compare the real and virtual scenarios.

In the third and last session, a series of questions were asked to facilitate reflection on the activity. This session was conducted online through the application of a *Google Forms* questionnaire (Q3 and Q2-B). Questions were asked to evaluate the usefulness of interactive experiences in the garden and the landscape as interdisciplinary educational resources in teacher training and analyze the impact the impact on learning and teaching skills.

### Measures

A mixed method was used in the study to better understand the research question so that the quantitative data could be supported by qualitative data for its interpretation (Creswell, 2014). The participants completed three questionnaires. The first (Q1) was composed of three tables for noting visual and auditory information on the landscape: Q1-T1: description of auditory elements and classification by parameters, Q1-T2: emotional perception of the landscape, and Q1-T3: musical recreation or recreating ambient sounds by means of musical instruments with selection of musical styles to reproduce landscapes in sound. The Q1 questionnaire was completed by the participants during the outdoor session (in stops made between the Faculty of Education and the garden).

The second questionnaire (Q2) was composed of nine open questions to assess the use of VR as a tool for recreation and visualization of landscapes (Q2-A), reflect on the relationship between the environment and health (Q2-C), and analyze the advantages, difficulties, and limitations of activities in the garden (Q2-B). The questions in Q2 are given in Table 3. The Q2 questionnaire was completed during the classroom session.

**TABLE 3:** Questions in qualitative Q2 questionnaire.

**Q2-A. On immersion in virtual landscape**	
P1	What elements can you see in the real and virtual landscapes? Any differences? What are they?
P2	What sensation does immersion give you?
P3	Do you think the virtual scenes objectively represent the reality you see?
**Q2-B. Analysis of use of garden as educational resource**	
P4	What aspects and advantages would you highlight?
P5	Do you think a garden is appropriate for primary teaching?
P6	Can you find any difficulties?
P7	How would you work with the soundscape in primary education?
**Q2-C. Reflections on the relationship between the environment and health**	
P8	What type of relationships exist between the environment and health?
P9	Did you find changes in the sounds during the itinerary? What were they? Positive or negative?

The third questionnaire (Q3), designed to analyze the constructs *perception of learning* and *acquisition of teaching competencies*, consisted of 18 items on a 10-point Likert scale. Perception of learning was analyzed through six items that included different aspects such as the disciplinary content, the usefulness of the teaching materials, the use of information and communication technologies (ICT) as a complementary resource, and the degree of interdisciplinarity of the knowledge achieved (Q3-A). The impact of the garden activities on the development of competencies was evaluated by means of 12 items that included the general and specific competencies that must be achieved by students in the Teaching Degree (Q3-B). The Q3 questionnaire was completed in the third session (online).

### Data Analysis

The Q1 and Q2 questionnaires were analyzed following the phenomenographic approach proposed by Marton (1988, 2015). According to Miles et al. (2014), in the qualitative approach, data collection focuses on the participants’ perspectives and points of view (emotions, priorities, experience, and meanings) and has an inductive rationale because it explores and describes reality in order to generate theoretical perspectives. The results of the Q1 questionnaire were organized into categories. As pointed out by Marton and Booth (1997), the definition of categories must meet a series of criteria: (a) each category is clearly related to the phenomenon under study; i.e., each one must tell us something different about the particular form of explaining the phenomenon; (b) the categories are in hierarchical order and so must progress from simple to complex relationships; (c) the system is simple; i.e., they can be explained by a reasonably small number of categories. If the category system meets the above criteria, it will be theoretically and pedagogically useful (Marton and Booth, 1997).

The answers to the Q3 questionnaire were analyzed by SPSS to obtain the descriptive statistics (mean and standard deviation). Cronbach α reliability coefficient was used to analyze its internal consistency. This assumes that the items measure dimensions of the same construct that are related to each other (Welch and Comer, 1988). This coefficient was applied to the set of items that evaluate the *acquisition of teaching competencies* construct (Q3-B) in a pilot sample of 61 students. However, it was not applied to Q3-A questionnaire because the items used to analyze the construct *perception of learning* measure different theoretical dimensions that are not interrelated (content, teaching materials, use of ICT, interdisciplinarity).

## Results

### Sensorial Landscape Perception

The sounds identified by the participants in Q1-T1 questionnaire were analyzed considering five parameters: *production* (source of the sound), *frequency* (number of times the sound is repeated), *intensity* (power of the sound), *sensation* (emotional perception), and *location* (distance to the point at which the sound is produced). A series of categories were defined for each parameter, and the sounds were classified (Dubois, 2000). In this way, each sound was described using five attributes or categories, one for each parameter. For example, the sound of water was classified into the following categories: natural production, continuous frequency, soft intensity, pleasant sensation, and close location (Table 4).

**TABLE 4:** Classification of ambient sounds according to five parameters, Q1-T1.

Parameter	Categories	Landscape		
		Faculty 65 dB	Urban 75 dB	Garden 45 dB
	
		Sounds	Sounds	Sounds
Production	Natural	–	Wind	Birds, wind, water, animals, leaves
	Human	Murmurs, voices, shouts, conversations	Voices	Steps, voices
	Technological	Elevator, doors, bell, music	Cars, whistles, bicycles	Tractor, cars, bicycles
Frequency	Continuous	Murmurs, conversation, music	Wind	Wind, water, leaves.
	Repetitive	Voices	Cars, whistles, voices	Cars, birds, steps, voices
	Once	Shouts, bell, elevator, doors	bicycles	Tractor, bicycles, animals
Intensity	Loud	Shouts, voices, bell	Cars, whistles, voices	Cars, tractor
	Soft	Murmurs, conversation, elevator, doors, music	Wind, bicycles	Wind, water, leaves, steps, voices, bicycles, birds, animals
Sensation	Pleasant	Conversation, music	Wind, bicycles	Wind, water, leaves, steps, voices, bicycles, birds, animals
	Unpleasant	Shouts, voices, murmurs, elevator, doors, bell	Cars, whistles, voices	Cars, tractor
Location	Distant	Shouts, elevator, doors	Whistles	Cars, birds, animals
	Near	Murmurs, conversation, bell	Cars, bicycles, voices	Water, leaves, steps, voices
	Medium	Voices, music	Wind	Wind, tractor, bicycles
**TSn**		8	5	10

*TSn, total sounds number in the landscape.*

The sound source (production) was selected to create the hierarchical system of categories and to classify the ambient sounds. According to Dubois et al. (2006), the identification of the source is the main signal for the formation of categories in the upper (soundscapes) and intermediate (natural and human sounds) levels of the taxonomy. Guastavino (2007) showed that 76% of the descriptions of soundscapes are based on sound sources. This is consistent with previous suggestions that, in this level of differentiation, sounds are typically classified by perceived similarities between sound sources rather than by abstract acoustic characteristics (Gaver, 1993; Houix et al., 2012). It also coincides with daily listening, which is mainly concerned with gathering information about sound sources (Gaver, 1993). Three categories were identified according to *production* parameter: *nature*, *human*, and *technological*, following the taxonomy proposed by Schafer (1993) and Bones et al. (2018). The *nature* category was divided into three subcategories: *animals*, *plants*, and *abiotic elements*. The *human* category was divided into two subcategories: *voices* and *music*, and *technology* category into two: *industrial* and *household*. The selected hierarchical category system therefore was composed of three categories and seven subcategories, and the sounds were classified (Table 5).

**TABLE 5:** Hierarchical classification of sounds according to production parameter.

Category	Subcategory		Landscape					
		Faculty		Urban		Garden	
		
		Sn	Sounds	Sn	Sounds	Sn	Sounds
Natural	Animals	0	–	0	–	2	Birds and animals
	Plants	0	–	0	–	1	Leaves
	Abiotic elements	0	–	1	Wind	2	Wind and water
Human	Voices	4	Murmurs, voices, shouts, and conversation	1	Voices	2	Steps and voices
	Music	1	Music	0	—	0	—
Technological	Industrial	0	–	3	Cars, whistles, and bicycles	3	Tractor, cars, and bicycles
	Household	3	Elevator, doors, and bell	0	–	0	–
**TSn**		8		5		10	

*Sn, sounds number by subcategory; TSn, total sounds number in the landscape.*

When classifying sounds according to their source, it is observed that a greater number of sound elements are perceived in the garden than in other landscapes. In addition, the garden has a greater diversity of sound elements than the rest of the landscapes and combines sounds from all three categories: natural, human, and technological. For instance, in the human landscape (inside the Faculty of Education), no natural sounds were perceived, whereas technological sounds predominate in the urban landscape.

In the analysis of the emotions generated by soundscapes, the expressions that described the emotional states were grouped into three categories: pleasant, neutral, and unpleasant (Q1-T2 questionnaire). The garden was shown as the landscape in which emotional perceptions are most positive because it received descriptions and expressions such as tranquility, peace, and freedom. These results are similar to those observed by Axelsson et al. (2010). According to Herranz-Pascual et al. (2019), the characteristics of the soundscape that contribute to well-being and the reduction of perceived stress are calm and tranquil. In this way, a positive affective response to natural open environments allows the individual to recover from fatigue and negative emotional states (Ulrich, 1981). The level of well-being was found to be very low or nil in the urban landscape, and most of the emotions were described as unpleasant. Table 6 shows the categorization of emotions in relation to well-being.

**TABLE 6:** Classification of emotions by level of well-being, Q1-T2.

Categories	Faculty		Urban		Garden	
	En	Emotions	En	Emotions	En	Emotions
Pleasant	2	Enjoyment and euphoria	0	–	8	Peace, calm, tranquility, relaxation, liberty, joy, serenity, and motivation
Neutral	2	Routine and boredom	1	Confusion	0	–
Unpleasant	4	Stress, exasperation, discomfort, and bother	4	Stress, exasperation, anxiety, and nervousness	0	–
**TEn**	8		5		8	

*En, emotions number by category; TEn, total emotions number in the landscape.*

The analysis of the relation between sound and music (Q1-T3) showed that the instruments selected to represent the urban landscape with most technological sounds were percussion and electronic instruments (electric guitars) with rock- and heavy metal–type music. In the garden landscape, they were stringed (violin, harp, and piano) and wind instruments (flute, harmonic, and clarinet), classical music, and jazz and blues. Inside the Faculty, sounds (voices and conversations) were related to string instruments (acoustic guitar), wind instruments (saxophone, tuba, trumpet, and trombone), and pop, blues, and classical music (Table 7).

**TABLE 7:** Musical recreation of landscape: instruments and musical styles, Q1-T3.

Landscape	Musical instruments	Musical styles
Faculty	Acoustic guitar, drums, saxophone, tuba, trombone, clarinet, and trumpet	Pop, blues, and classical
Urban	Drums, electric guitar, tuba, oboe, and trombone	Rock, techno, heavy metal, and reggae
Garden	Violin, flute, clarinet, triangle, harp, guitar, harmonica, xylophone, clarinet, cymbals, piano, and transverse flute	Classical, chill-out, reggae, ambient, jazz, and blues

In the second session in the classroom with VR, the results of questions Q2-A were analyzed: *What elements can you see in the virtual and real scenarios? Are there any differences? What are they?* (P1), and *What sensations do you get from the immersion experience?* (P2). More than 90% of the participants indicated positive sensations and pleasant and could identify most of the visual and sound elements in the garden. The descriptions of the landscapes were detailed and similar to those obtained in the first session in the garden. Some participants indicated a certain confusion and dizziness during immersion due to the distortion between the real and virtual scenarios. This distortion can be avoided if the virtual experience is carried out in the same place as the recording, so that the contrast between virtual and real is reduced.

In answer to the question*: Do the virtual scenes objectively represent the reality you can see*? (P3) 85% answered positively but depth was not clearly perceived: “The avocado video and the one of the donkey gave me the sensation of not being real since I could not see the depth clearly.” However, they showed great interest in virtual immersion to foment listening and avoid the acoustic contamination in real landscapes: “I like the VR headset more than the naked eye due to the acoustic contamination.” They also highlighted the use of VR as a tool to observe temporary changes in the landscape: “It is curious; it lets you see something that was there in the past but not in the present.”

### The Relationship Between the Environment and Health

The study of the garden’s soundscape produced a series of reflections on the relationship between the environment and health. On the one hand, during the outdoor session, the participants measured the sound intensity in order to explore the variation in the noise level between the city and the garden. The analysis of the Q1-T1 questionnaire shows that the garden and its landscape have the lowest level of sound intensity (≤45 dB), whereas the highest is found in the urban environment (≥75 dB). Because at values higher than 70 dB you can feel discomfort and pain in the ear canal, it is confirmed that the urban landscape can generate levels of noise pollution that must be controlled to avoid human health risks. There is also an indirect relationship between the number and diversity of sounds perceived in each landscape, and the sound intensity is observed, so that greater intensity means a reduced ability to detect and identify sounds due to interference. This indicates that the garden has a buffering function against noise pollution and favors the listening of the healthy environment.

On the other hand, the analysis of the answers to Q2-C offered interesting opinions on this relationship and its importance. The following are some reflections that show students are capable of: (1) recognizing problematic situations [“Sometimes we don’t give importance to our auditory system (loud music, headphones…).” “It is important to know the situations that we have experienced because we can see the situations that can harm us.” “In the city, there is a lot of noise, and this can harm people’s health”]; and (2) acting on the problem (“We should be more conscious of it and take care of ourselves”). On the other hand, some reflections show that they are able to identify the healthiness of the environments and their differences. “It is healthier to live in the country because there is significantly less noise pollution.” “The sound level varies according to the place. We verified this when we measured it in the Faculty of Education and in the garden. We can conclude that there may be noise pollution in the city.”

### The Garden and Landscape as an Educational Resource

The results of the Q3 questionnaire showed the garden and landscape possibilities as an educational resource for learning the contents of subjects and acquiring the teaching competencies included in the Primary Teacher’s Degree syllabus. The first step analyzed the impact on learning through six items that were assessed on a scale of 0–10 (Q3-A). The results showed that the garden favored interdisciplinary aspects and the overall development of knowledge and improved the learning of contents (Table 8). The students positively evaluated the use of ICT as resource for learning (8.6). The didactic materials used during the activity were scored lower than the other aspects (7.5), probably because they were designed as self-learning practices, and students are not used to this type of activities. This shows the need to go on encouraging self-learning.

**TABLE 8:** Students’ perception of the impact of the garden activity on content learning, Q3-A.

Impact on learning	Mean	*SD*
	
(Likert 0–10: 0 = absolutely disagree, 10 = completely agree)	(*n* = 418)	
Relation with disciplinary content	8.0	1.23
Didactic materials used	7.5	1.05
Accessibility of ICT resources	8.0	1.51
Usefulness of ICT resources for learning	8.6	1.34
Learning improvement	8.4	1.01
Degree of interdisciplinarity and overall knowledge development	9.4	0.97

The Q3-B questionnaire’s internal consistency was analyzed by Cronbach α and obtained a value of 0.81, meaning high consistency and suitability for measuring *acquisition of teaching competence.* The mean results indicate good student perception of the use of the garden to develop professional teaching competencies (Table 9). The most developed competencies within the garden activity are those related to cooperative work, lifelong learning, and the design and application of educational strategies (GC5, GC6, GC9, GC10, and GC11). The students show a positive perception of the acquisition of scientific competencies and highlight problem solving through scientific reasoning (SC122 and SC124). They also consider that this type of educational projects promotes interest and respect for the environment and health. In general, they perceive very positively the level of teaching competencies achieved. Competencies GC4 and GC12, referring to knowledge transfer in everyday life and the development of argumentative and critical faculties, scored lowest. These results again reinforce the need to promote self-learning and students’ autonomous capacity in the educational process. As regards the use of TIC, the results showed low students’ perception, and this item had the widest point dispersion.

**TABLE 9:** Usefulness of garden and landscape for acquiring teaching competencies, Q3-B.

General competence (GC) and specific competencies (SC)	Media	*SD*
	
(Official syllabus for Primary Teacher’s Degree—UV)	(*n* = 418)	
GC3. Expert use of ICT as habitual work tools	6.80	2.26
GC4. Critically analyze and incorporate relevant social questions as they affect education at home and at school	7.98	1.50
GC5. Promote individual and group work	8.95	1.06
GC6. Assume that teaching should keep pace with scientific, educational, and social changes	8.70	1.09
GC9. Design, plan, and evaluate teaching–learning activities	8.72	1.11
GC10. Work in team with other professionals in planning class and extracurricular activities	8.67	1.42
GC11. Apply basic educational research techniques	8.62	1.05
GC12. Comprehend that systematic observation is a basic instrument for reflecting on practice and reality and contribute to educational innovation	7.92	1.97
SC122. Know, develop, and evaluate the curriculum in sciences and promote the acquisition of basic skills	8.15	1.35
SC124. Propose everyday problem-solving activities using scientific language and reasoning	8.52	1.47
SC127. Promote interest and respect for the environment and health through educational projects	9.52	0.72
Level of acquisition of professional teaching competence	8.62	1.34

The analysis of the answers to Q2-B questionnaire showed that most of the students consider the activity in the garden and the landscape as an educational strategy that favors the integration of theory with practice (96%). Moreover, 93% think that sensory interaction with the natural environment promotes environmental attitudes and respect for nature, 87% observe that sensory experiences contribute to a better understanding of environmental problems, 85% indicate that the theoretical contents are better understood when experimenting on the environment, and 78% believe that these types of activities are suitable for implementation in primary school as they are interactive activities that promote reflection on real situations. Regarding the difficulties, most of them (79%) consider that the trips to the natural environment require more teacher coordination than carrying out activities in the classroom. They also note that travel time and distance might limit these activities.

## Discussion

Sensorial ambient perception was used as a tool to recognize elements in the garden and its surroundings with an emphasis on auditory and visual phenomena as a support for understanding the agroecosystem and its impact on health and artistic expression. This research project on didactic experiences in natural surroundings used both quantitative and qualitative methods plus a phenomenological analysis in a mixed approach. A hierarchical system was established to group the perceived ambient sounds by the source of production in three categories and seven subcategories similar to those proposed in Bones et al. (2018) and in line with the study by Gaver (1993) on the consistence of the origin as the principal parameter in everyday hearing and the construction of categories in the soundscape. A study was also carried out on the synergy between the experimental sciences and artistic studies by examining the emotions and the musical recreation of natural and human landscapes. This approach showed that the garden and landscape are a first-order didactic resource for training teachers, in agreement with the findings of Delgado-Huertos (2015).

The quantitative study was in the form of a questionnaire on the impact of the garden on teachers’ training, and two constructs were analyzed: *perception of learning* and *acquisition of teaching competencies*. The results show that the garden is a good approach for teaching subject contents and developing competencies in student teachers (Cantó et al., 2013; Ceballos et al., 2014; Aragón, 2017; Eugenio et al., 2019). The interactive experiences of students in the garden and its surrounding landscape promote the integration of theoretical content with practice in order to effectively and cooperatively address the resolution of scientific, social, and cultural problems. This innovative proposal seeks improvement in the acquisition of professional skills through direct contact with nature (Parra et al., 2019). Some of the results reveal some difficulties of the participants in relation to autonomous learning and how informal learning contexts, activities in the field, and video recordings (360° virtual immersion) can contribute to improving autonomous learning (Sáez and Cortés, 2013).

After this outdoor experience, changes in the perception of students about environmental problems and their influence on health were assessed, and an improvement in the understanding of environmental processes was found. The high score obtained by competence CE 127 should also be emphasized. This reinforces the idea of the role of activities in the garden and its surroundings as promoters of environmental appreciation and the importance of nature (Evans et al., 2018; Sobko et al., 2018). The poor acquisition of digital competencies may be due to the students’ differences in the use of ICT. Thus, further research on the incorporation of these technologies in teaching is required.

These results highlight the need to design teaching models with a holistic approach that allows the formation of a critical and committed citizenship (UNESCO, 2018). This new model should encourage the acquisition of teaching competencies by preservice teachers and be oriented toward professionalization, as has been recommended by Alsina (2013) and Bolarín and Moreno (2015).

The study shows that experimentation and a holistic approach to the garden environment and the soundscape connect students with physical reality, give them improved sensorial and emotional perceptions, and generate proenvironmental attitudes (Hurtado et al., 2018a). It is therefore fundamental to continue investigating and implementing educational strategies that put the spotlight on the students themselves and integrate theory and practice. In this regard, we can mention here that the use of the garden and landscape is a first-order pedagogical model for the studies of future teachers and is along the same lines as other recent studies (Ceballos et al., 2014; Delgado-Huertos, 2015; Eugenio and Aragón, 2016; Eugenio et al., 2019).

## Conclusion

This learning garden study shows that nature is connected to cognitive, emotional, and attitudinal aspects and that the landscape can be perceived through the senses. This indicates that visual and auditory perception of the landscape is a first-order resource for approaching the natural, social, and cultural environments by a holistic, interdisciplinary, and experimental method. With this idea in mind, the usefulness of the garden as a teaching model for training teachers has many possibilities, especially the learning of the contents (science, music, painting) and generating positive attitudes to the natural environment. Among these, the opportunity to generate synergies between the scientific and artistic–musical disciplines stands out as the basis for a better understanding of natural phenomena and their relationship with human beings. Moreover, a high degree of satisfaction with the methodology used when working in the garden can be inferred from the participating students’ opinions. They seem to recognize the positive experience of feeling like teachers, because these educational situations allow them to reflect on the work of a teacher within the university context and in relation to the acquisition and transmission of values linked to sustainable development.

Without any doubt, this study has shown the need for a change of the educational model toward increased student autonomy for the effective development of professional teaching competencies. Those competencies that allow the integrated resolution of problems (situation analysis, anticipatory thinking, education in values, and interpersonal and strategic skills oriented to action) are particularly important for sustainable development. However, the implementation of this type of initiative in a university context requires complex coordination between teachers and very different areas of knowledge.

In this preliminary phase of the investigation, the effect of the participants’ gender on sensory perception has not been taken into account. However, some studies indicate the existence of representations and uses of the senses that include gender-differentiated forms of perception (Sabido, 2016). Therefore, it would be interesting to analyze these possible differences in the sample of this study (70.3% women and 29.7% men). Neither have other variables been considered, such as age, the subject being studied, and the training itinerary. This multifactor analysis is part of an ongoing quantitative study. On the other hand, we have to take into account that this study has been carried out in the surroundings of the *Huerta de Valencia* with a very specific landscape. Therefore, when replicating the research, we may have some difficulties in finding similar environments. However, we consider that this is an opportunity to analyze the relationship between the garden and its surroundings. Therefore, it would be interesting to replicate the research with learning gardens located in different contexts and landscapes.

Finally, the learning garden and its landscape can offer many educational approaches and permit the integration of all the subjects in the curriculum in a permanent lifelong teaching–learning process that improves the capacity to respond to social, environmental, and cultural problems consistently, critically, and rigorously in order to achieve a sustainable and inclusive world.

## Data Availability Statement

The datasets presented in this article are not readily available because raw data is being used in subsequent studies. Requests to access the datasets should be directed to AH-S, amparo.hurtado@uv.es.

## Ethics Statement

Ethical review and approval was not required for the study on human participants in accordance with the local legislation and institutional requirements. The participants provided their written informed consent to participate in this study.

## Author Contributions

AH-S and AB-N designed the study. AH-S, AB-N, and SM-G collected the data. AH-S, AB-N, and PM-L analyzed the data. All authors wrote the manuscript.

## Conflict of Interest

The authors declare that the research was conducted in the absence of any commercial or financial relationships that could be construed as a potential conflict of interest.

## Footnotes

1 https://cutt.ly/raf7BbG
