# Supplementary material for: The Garden and Landscape as an Interdisciplinary Resource Between Experimental Science and Artistic–Musical Expression: Analysis of Competence Development in Student Teachers
Source: Front Psychol. 2020 Sep 4;11:2163. doi: 10.3389/fpsyg.2020.02163 (PMC7499801; doi:10.3389/fpsyg.2020.02163)
Supplement: Supplementary file 1 [file Table_1.DOCX]

## **Activity Session 1. The orchard and the soundscape Questionnaire Q1**

## Part 1: Tour of Faculty-Urban-Garden (individual)

Student_____________________________________________________________________

Subject____________________________________________________________________

Female Male Age________

University access studies _________________________________________________

1. **Complete the tables below:**

| **Limit** | **Effect to be avoided or situation in which it applies** |
| --- | --- |
| 100 - 130 dBA | Auditory discomfort |
| 130 - 140 dBA | Risk of physical damage (e.g. perforated eardrum) |
| 130 dBA | Severe pain |
| 70 dBA L_eq24_ | Insignificant hearing damage |

| **Sound intensity level** | |
| --- | --- |
| 200 dB | Atomic bomb |
| 180 dB | Krakatoa Volcano explosion (160 km away) Rocket in take off |
| 142.2 dB | Guinness World Record for noise in a stadium |
| 140 dB | Pain threshold. Formula 1 car |
| 130 dB | Plane in take-off |
| 120 dB | Aircraft engine running. Fireworks. |
| 110 dB | Concert. Civic act |
| 100 dB | Electril drill |
| 90 dB | Traffic |
| 80 dB | Train |
| 70 dB | Vacuum cleaner |
| 50/60 dB | Clustering. Dishwasher |
| 40 dB | Conversation |
| 20 dB | Library |
| 10 dB | quiet breathing |
| 0 dB | hearing threshold |

L_eq24_: Equvialent level for 24 hours

**Sound level measurements. Take three measurements and calculate the average:**

| **Nº** | **Landscape** | **dBA** |
| --- | --- | --- |
| 1.1 | Faculty |  |
| 1.2 | Faculty |  |
| 1.3 | Faculty |  |
| Promedio | Faculty |  |
| 2.1 | Urban |  |
| 2.2 | Urban |  |
| 2.3 | Urban |  |
| Promedio | Urban |  |
| 3.1 | Garden |  |
| 3.2 | Garden |  |
| 3.3 | Garden |  |
| Promedio | Garden |  |

Reflect on the results obtained taking into account their correspondence with the sound intensity levels in the attached table.

____________________________________________________________________________________________________________________________________________

**Stop 1. Human landscape (Inside the Faculty)**

Table 1.1 Classify the sound elements you perceive based on these parameters (mark with a cross):

|  | ***PRODUCTION*** | | | ***FREQUENCY*** | | | ***INTENSITY*** | | ***SENSATION*** | | ***LOCATION*** | | |
| --- | --- | --- | --- | --- | --- | --- | --- | --- | --- | --- | --- | --- | --- |
| ***Sound*** | *Natural* | *Human* | *Technological* | *Continuous* | *Repetitive* | *Once* | *Loud* | *Soft* | *Pleasant* | *Unpleasant* | *Distan* | *Near* | *Medium* |
|  |  |  |  |  |  |  |  |  |  |  |  |  |  |
|  |  |  |  |  |  |  |  |  |  |  |  |  |  |

Table 1.2 Relate sounds to musical instruments. With which musical instruments would you recreate the perceived sounds?

| **Sound** | **Stringed instruments** | **Wind instruments** | **Percussion instruments** |
| --- | --- | --- | --- |
|  |  |  |  |
|  |  |  |  |

**Stop 2. Urban landscape (Street with traffic)**

Table 2.1 Classify the sound elements you perceive based on these parameters (mark with a cross):

|  | ***PRODUCTION*** | | | ***FREQUENCY*** | | | ***INTENSITY*** | | ***SENSATION*** | | ***LOCATION*** | | |
| --- | --- | --- | --- | --- | --- | --- | --- | --- | --- | --- | --- | --- | --- |
| ***Sound*** | *Natural* | *Human* | *Technological* | *Continuous* | *Repetitive* | *Once* | *Loud* | *Soft* | *Pleasant* | *Unpleasant* | *Distan* | *Near* | *Medium* |
|  |  |  |  |  |  |  |  |  |  |  |  |  |  |
|  |  |  |  |  |  |  |  |  |  |  |  |  |  |

Table 2.2 Relate sounds to musical instruments. With which musical instruments would you recreate the perceived sounds?

| **Sound** | **Stringed instruments** | **Wind instruments** | **Percussion instruments** |
| --- | --- | --- | --- |
|  |  |  |  |
|  |  |  |  |

**Stop 3. Agricultural landscape (Garden and *Huerta de Valencia*)**

Table 3.1 Classify the sound elements you perceive based on these parameters (mark with a cross):

|  | ***PRODUCTION*** | | | ***FREQUENCY*** | | | ***INTENSITY*** | | ***SENSATION*** | | ***LOCATION*** | | |
| --- | --- | --- | --- | --- | --- | --- | --- | --- | --- | --- | --- | --- | --- |
| ***Sound*** | *Natural* | *Human* | *Technological* | *Continuous* | *Repetitive* | *Once* | *Loud* | *Soft* | *Pleasant* | *Unpleasant* | *Distan* | *Near* | *Medium* |
|  |  |  |  |  |  |  |  |  |  |  |  |  |  |
|  |  |  |  |  |  |  |  |  |  |  |  |  |  |

Table 3.2 Relate sounds to musical instruments. With which musical instruments would you recreate the perceived sounds?

| **Sound** | **Stringed instruments** | **Wind instruments** | **Percussion instruments** |
| --- | --- | --- | --- |
|  |  |  |  |
|  |  |  |  |

1. **Musical recreation of the landscape**

Table 4. Imagine that you want to recreate these soundscapes. With which music/music would you relate each landscape?

| **Soundscape** | **Musical Styles** |
| --- | --- |
| **Faculty** |  |
| **Urban** |  |
| **Garden** |  |

1. **Emotional landscape perception**

Table 5 What sensations do you perceive in the landscape? Describe with adjectives the emotions that each soundscape produces in you

| **Soundscape** | **Emotions** |
| --- | --- |
| **Faculty** |  |
| **Urban** |  |
| **Garden** |  |

Relate perceived emotions to the level of well-being you think each landscape offers

__________________________________________________________________________________________________________________________________________________________________________________________________________________

Finally, reflect on the experience of sensory perception you have made

________________________________________________________________________________________________________________________________________________________________________________________________________________________________________________________________________________________

## **Part 2: Observations in the Garden (group)**

Team members (max. 4)

__________________________________________________________________________________________________________________________________________________________

Subject____________________________________________________________________

1. **Answer the following questions:**

Describe the crops in the garden and their state of conservation

_______________________________________________________________________________________________________________________________________________________________________________________________________________________________________

What environmental problems have you detected in the Garden and its surroundings (landscape)?

__________________________________________________________________________________________________________________________________________________________

Regarding noise pollution... What sound elements in the garden do you think produce noise pollution? What is its origin? Indicate possible solutions

____________________________________________________________________________________________________________________________________________________________________________________________________________________________________________________________________________________________________________________

Other interesting comments

____________________________________________________________________________________________________________________________________________________________________________________________________________________________________________________________________________________________________________________
